# Age equality in UK healthcare policy and decision-making: a scoping review

**DOI:** 10.1186/s12910-026-01508-1

**Published:** 2026-06-24

**Authors:** Sapfo Lignou, Rachel Thompson, James Hart, Cai Heath

**Affiliations:** Ethox Centre, Nuffield Department of Population Health, University of Oxford, Oxford, Big Data Institute, Li Ka Shing Centre for Health Information and Discovery, Oxford, OX3 7LF UK

**Keywords:** Age equality, Age discrimination, Healthcare decision-making, Policy, Law, Clinical guidance, Age-groups, Equality act, Commissioning, Health inequalities

## Abstract

**Background:**

Age equality was enshrined in UK law by the Equality Act of 2010, which defined age as a protected characteristic and age discrimination unlawful, unless objectively justified. To date relatively little attention has been paid to how this legal requirement is interpreted and applied in healthcare. Such a lack of clarity raises significant legal and ethical questions regarding how age affects access to and provision of care. This scoping review aims to address this gap. It examines how age and age groups are considered in healthcare policies and decision-making processes and how age equality duties are understood and implemented in practice.

**Methods:**

The scoping review draws on national and regional policy and decision-making documents, and relevant academic literature. It has two main objectives: first, to examine the explicit and implicit ways age influences healthcare provision. Second, to assess how different age-groups are represented or omitted in healthcare decision-making processes that determine access to care. Attention is given to age-based distinctions and omissions, their stated justifications or their absence in policy, and healthcare decision-making.

**Results:**

Our findings indicate that despite legal protections, age equality is not yet consistently embedded in healthcare policy or practice with significant unresolved ethical tensions in approaches to age-equality. We identified gaps between stated policy commitments and implementation, limited attention to age-related inequalities and little clarity on when such inequalities may constitute inequities or raise concerns about unlawful discrimination. These include a lack of evidence for systematic monitoring of age-related inequalities and potential inequities, and lack of practical guidance to support healthcare decision-makers in meeting their equality duties.

**Conclusions:**

It remains unclear whether and how key ethical tensions in the pursuit of age equality are resolved by decision-makers. To support a more consistent and transparent approach to age equality in healthcare, efforts should focus on strengthened decision-making processes and clearer operational guidance. The routine use of age-sensitive equality impact assessments across services could enable a more systematic identification of age-related health inequalities and inequities that may be overlooked across the lifecourse.

**Supplementary Information:**

The online version contains supplementary material available at https://doi.org/10.1186/s12910-026-01508-1.

## Background

In the United Kingdom, age equality is governed primarily by the Equality Act 2010. According to the Act, age discrimination can be either direct or indirect. Direct age discrimination occurs when an individual is treated less favourably because of their age, whilst indirect age discrimination occurs when a universal rule or policy would disadvantage one age group in comparison to others. The Act makes age discrimination in the provision of services and public functions unlawful, unless such treatment can be justified as a “proportionate means of achieving a legitimate aim” (Equality Act 2010, Sect. 13(2) and 19). This proportionality test does not usually apply to direct discrimination but does in the case of age. Thus, unlike most other protected characteristics, the Act permits age to be taken into account within defined limits, reflecting both its potential relevance and the risk of harm from its unjustified use in decision-making.

Even at the time of its development, concerns were raised about how age equality would operate in practice. Preparatory work for the Act drew attention to the ways in which routine decision-making tools could reinforce disadvantage without deliberate intent. In particular, the Carruthers review (2009) warned that seemingly neutral policies (such as in the Quality Adjusted Life Years (QALYs) formulas or social care charges) could produce indirect age discrimination, which can be as harmful in its effects as direct discrimination. The review recommended audits and clear action to promote age equality. Subsequent guidance from the Department of Health and Government Equalities Office (2012) endorsed the positive use of age “in providing, commissioning and planning services” ([26], p. 5) while clarifying that such measures should not replace individual assessments of need (ibid, p. 8).

Later regulatory frameworks reinforce that any use of age in healthcare decisions must satisfy the legal test of proportionality and legitimate aim (Government Equalities Office & Equality and Human Rights Commission 2015). These frameworks explicitly reject both blanket age-based exemptions and “age-blind” approaches that overlook age-related needs [45]. The NHS Act 2006 (as amended by both the Health and Social Care Act 2012 and the Health and Care Act 2022 to clarify equalities duties) along with the Equality and Human Rights Commission Code of Practice (2011) and its updated guidance ([35] 2023 [46, 138]) require service providers and local authorities to meet their Public Sector Equality duties by giving ‘due regard’ to how their decisions affect different groups, including people of different ages [139].

The legal and regulatory framework establishes clear principles regarding the use of age and the prohibition of age discrimination in healthcare provision. However, translating these principles into healthcare policy and decision-making is more complex. The law allows age to be considered within defined limits but offers little clarity on how these limits should be interpreted and applied in practice (Hilton 2018). As a result, healthcare policies and decision-making frameworks must balance legal requirements with practical challenges of service and care delivery, a challenge which is further compounded by limited resources, increasing health inequalities and competing demands within the healthcare system [24]. As not every need can be met, difficult decisions regarding care prioritisation are unavoidable. Choices regarding which treatments to fund, how care should be delivered and who has access to it, affect people of all ages and in different ways. Ensuring therefore that equality duties are properly considered and consistently applied is challenging yet an important safeguard against arbitrary or unjustified treatment when decisions about funding, organisation, and delivery of care are made.

Despite this extensive legal and policy framework, to date there is limited understanding of how the principles of age equality are interpreted and practically applied across different levels of the healthcare system. This gap is striking given the long-standing recognition of the difficulty in translating equality law into practice [173]. Little is known about how age actually influences decision-making across the healthcare system. Research on this issue is fragmented, focusing on specific areas, such as the economic implications of an ageing population for healthcare expenditure [156], or limited to specific clinical settings, guidelines or populations [37, 71, 146, 148, 152, 157]. When age is explicitly considered, this is to examine its role as an eligibility criterion or barrier for treatment [55] and often for particular procedures, such as life-saving or fertility treatments or cancer screening [145, 166, 170]. Although this evidence is valuable, it offers limited insight into the ways in which age is operationalised in the decisions that determine healthcare provision as a whole.

This scoping review aims to address this gap. It has two main objectives. First, to understand how age features into current policies and decision-making documents which determine the way in which healthcare is provided. Second, to explore how different age groups are represented or omitted within frameworks that determine access to care. The review provides evidence on where age-based distinctions occur, and their justifications or their absence, and concerns raised regarding their impacts on people at different stages of life. It also identifies ethical tensions that arise when equality duties are implemented in policy and practice. We conclude that inconsistencies in procedural approaches, rather than clear instances of unlawful discrimination, indicate the need for stronger normative and ethical guidance to support age equality in healthcare.

## Methods

### Rationale

Scoping studies as defined by Arksey and O’Malley provide a structured, but more rapid and less restrictive, alternative to a systematic review of the literature ([143], p6). The development of this scoping review followed an iterative process which aimed to examine the range, nature and extent of literature in this area, including national and regional policy guidance and healthcare decision-making frameworks, and to identify gaps in the available evidence. This methodology was selected over a full systematic review as its purpose was to explore the available knowledge regarding age considerations in the UK healthcare decision making and policy, rather than answer a narrowly defined research question with a fixed set of studies. The breadth and heterogeneity of the literature and the exploratory nature of the research questions meant that a scoping review with a narrative synthesis was the most appropriate method to represent the evidence and identify gaps in knowledge pertaining to how age and age-groups are reflected in healthcare policy and decision-making.

### Search strategy

Based on the recommendations for scoping reviews from Peters et al. 2020, the search strategy was developed using inclusion criteria based on the “population-concept-context” framework [167, 168]. The PRISMA-ScR checklist was used as a reference throughout the search and data extraction process [172]. Authors (SL, CH, JH) developed the search strategy with support from Nia Roberts, Senior Outreach Librarian at the Bodleian Health Care Libraries, University of Oxford, to identify records containing the key concepts of age-related decision making, healthcare policy and equality. The authors agreed a list of key terms and performed an initial search of the Medline database, the results of which were reviewed by title and abstract to test the relevance of the search criteria before broadening to other databases.

The initial search was limited to studies and documents published between January 2009 and October 2024 to capture the period immediately preceding and following the introduction of the UK Equality Act (April 2010). A structured search in international literature was conducted using the following databases: Medline (OvidSP), HMI (OvidSP), EconLit [EBSCOHost], PAIS International, Policy File, ASSIA, Sociological Abstracts & Sociology Database [Proquest] and Overton. A UK-specific geographical search filter developed by information specialists at the National Institute for Health and Care Excellence (NICE) was used to refine the search [144]. Only articles written in English were included. Records from the different bibliographic databases were imported to Covidence then titles and abstracts of studies were screened for eligibility, using the a priori inclusion criteria described in Table 1.

**Table 1:** Scoping review inclusion criteria

Inclusion criteria	Definition	Synonyms and key search terms
Concept	Age-related publicly-funded healthcare resource allocation and decision-making	Age factors, age-related, age-based, life, health policy, delivery of health care, decision-making, priorities, National Health Service, equity, ethics
Context	United Kingdom, including devolved and regional healthcare in England, Northern Ireland, Scotland and Wales.	Limit = United Kingdom, England, Northern Ireland, Scotland, Wales
Date limits	Documents from the past 16 years to ensure relevance to current practices and policies, specifically the Equality Act of 2010.	Limit to year = “2009–2025”
Language	English language only	Limit = English language
Population	All conditions and cohorts considered	
Type of literature	Any published literature, both academic and non-academic. This includes peer-reviewed academic journal papers, systematic reviews, and guidelines. This includes multi-country studies and systematic reviews that include the United Kingdom.	

Manual searches were also conducted, we searched Google on 25th October 2024, screening the first 100 results for each search string, if the first 50 references were not relevant we stopped. The search strategy included title, abstract, author keywords and subject headings for our key concepts of age-related decision making, health policy and ethics. Additional manual searches were conducted in an iterative team approach that met regularly to discuss inclusion and exclusion criteria,and were guided by the contents and gaps in the initial academic searches [158]. This approach particularly aimed to identify grey literature and policy documentation publicly available on UK parliament, NHS, and government webpages, websites of devolved nations were also checked. The manual searches were informed by consultation with legal experts and healthcare commissioners to help identify documents used in healthcare decision-making. This consultative process extended the duration of the searches but strengthened the relevance of our results to policy and commissioning contexts. Other documents were identified in the references of previously extracted literature, in particular from parliamentary and governmental policy documents. Some of these documents were no longer openly accessible, so the Internet Archive and “Wayback Machine” were used to find them. That these papers were not otherwise accessible, does suggest, however, that other older policy documents may have since been deleted and not identified in our searches. The academic searches were updated in September 2025 once the manual searches were agreed to be saturated.

Duplicates were removed at the title and abstract screening stage. The PRISMA flowchart in Appendix 1 shows the selection process resulting in the included studies.

### Data extraction and analysis

The search provided 826 unique records to be screened and resulted in the inclusion of 138 documents that matched the criteria for the search.

Titles and abstracts were independently screened by RT, CH, JH and SL (RT + CH dual screened 20 studies to test inclusion criteria) and ‘borderline’ cases agreed by a consensus (SL, CH, JH, RT). The update to the review was conducted by CH, SL and JH in October 2025. Documents selected for full-text review (240) were checked for relevance based on the methodology for scoping reviews (Akiyu et al.).

Following this process,138 studies were included for data extraction. Data were extracted from the included studies using a data charting form (Covidence Extraction 2) designed by consensus and tested on several evidence types before commencing extraction of the included documents. The authors used a system of tagging papers by age groups and medical conditions, as well as inductively noting themes arising in the data and recording illustrative quotes from each text. RT and JH dual-reviewed during the full-text review process, clustering themes by age group. Regular meetings with the wider review team were held to resolve differences in categorisation and to ensure fair description.

RT, CH, JH, SL each reviewed a selection of papers included for full text reading, and extracted the following information: authors, title and year of publication, country, aims, services and type of service users, method and type (e.g., qualitative, quantitative, policy brief), findings/results related to how age and age groups feature in UK healthcare decision-making policy and documents, and main conclusions. A second researcher checked the extracted data and findings were combined in the same format with literature from the supplementary search of grey literature to support the thematic analysis. All results were collected in a Zotero library to record the findings and for data management and referencing processes.

Recurring themes in the findings identified throughout the review process were contemporaneously noted by researchers. The summary of findings for each article was then re-read and coded by three reviewers (RT, SL, JH) into the common themes, this process was repeated when the searches were updated by CH, JH and SL. These were discussed with the project team and agreed themes are reported in the results section. Interpretation of the reviewed documents focused on ensuring that recorded themes and comments were descriptive and not based on the opinion of the authors or broader context. The categorisation of findings by age-groups reflects patterns in the available literature and is used as an analytical tool to organise findings rather than to impose predefined normative distinctions.

## Results

### Description of included studies

Further presentation of the 138 documents included in the scoping review is organised based on the key themes found in our analysis. The academic database searches produced 63 results, while 75 papers were found manually. To break down documents broadly by type: 78 were non-academic policy documents, reports, legal documents, and guidance; while 60 were academic journal articles.

The 78 non-academic documents included 33 policy documents and government-issued guidance, 23 reports from both civil society and government sources, 13 ethical frameworks and priority setting guidance documents, 5 legal documents including UK legislation, and 4 briefing notes and commentaries. The documents were mainly UK-wide or England-specific in terms of scope. Of the ethical frameworks and guidance included in the analysis 8 were from regional NHS England health bodies.

In terms of methodology of the 60 journal articles included in the review, 22 of the included articles used empirical research methods (quantitative, cohort and cross-sectional study designs); 16 articles were qualitative designs, 21 were commentaries or conceptual pieces, 4 were systematic, scoping and case note reviews, and 2 used mixed methods approaches.

Across the entire review, the studies included represented a wide range of health disciplines, many documents were broad in nature across health domains and healthcare equity and age, however the following areas were included multiple times as the main focus of documents: the care and health of older people (*n* = 24); paediatric services and the transition into adult services (*n* = 19); mental health including neurodevelopmental conditions such as ADHD and autism (*n* = 18); non-communicable diseases including cancer, stroke and cardiovascular disease (*n* = 18); women’s and reproductive health (*n* = 11); infectious disease and pandemics (*n* = 7). The studies on mental health and cancer screening particularly had the strongest empirical coverage with a range of quantitative and qualitative studies and longitudinal data included along with several scoping and systematics reviews. Conversely, documents on women’s health and reproductive health were a mixture of empirical work and conceptual or commentary documents, indicating that there could be a gap for more empirical work on healthcare decision-making in this area.

To present how age and age groups feature in healthcare decision-making documents, findings were organised by commonly used age-bands in the literature: children and young people (0- ~ 21); working age adult (18- ~ 65); older adult (65 +). These distinctions are used descriptively. Some overlap between bands is unavoidable; for example, the category ‘working age adult’ includes young people (18–21) but was the most frequently used term in our sources to describe adults aged 18–65 and is used consistently throughout our reporting. Within and across these groups, we analyse how age is used as an explicit or implicit criterion, an omitted or restricted characteristic and how this relates to observed inequalities and potential inequities. Several cross-cutting themes emerged across all age-groups around national laws, clinical guidance, resource allocation and patient prioritisation; these are presented separately below.

While the legal framework focuses on discrimination, discussion on age equality also involves questions related to age-related inequalities and inequities. Throughout this review we use the following terms: i) inequality to refer to observed differences in healthcare provision or outcomes between age groups, ii) inequity to refer to inequalities that are avoidable and unfair, iii) discrimination, in its legal sense, to describe age-based differential treatment that may constitute direct or indirect discrimination under the Equality Act 2010, unless it can be objectively justified as a proportionate means of achieving a legitimate aim. Where relevant, we refer to practices *that may raise or have raised concerns about discrimination,* without implying that they meet the legal threshold. The analysis does not seek to determine whether specific healthcare practices are legally discriminatory but to examine how they relate to observed inequalities and potential inequities within the framework of age equality duties.

### Age in Healthcare decision-making and policy: cross-cutting perspective

#### Age in healthcare planning and funding formulas

In the NHS Long Term Plan (2019), primarily covering the NHS in England, references to age are largely framed in terms of service planning and organisation including sections on “Ageing Well” (NHS & UK Government, 2019b, p. 16–18) and “Starting Well” (NHS & UK Government 2019 p. 45–55) that set out priorities for older people and children. The new 10 Year Health Plan for England (DHSC & PMO 2025) also addresses age-related health needs and priorities, such as mental health support for children and young people. Both documents discuss health inequalities broadly and treat age as a demographic characteristic, but neither makes explicit reference to age equality duties or discrimination concerns in the provision of healthcare.

Age is also explicitly considered in decision-making processes determining how healthcare resources are allocated and clinical priorities are set. In England the Weighted Capitation Formula determines target funding for each local area and gives extra weight to older populations on the grounds that “all else equal, areas with older populations typically have a higher need per head” ([87], pp.9–10). However, a substantial body of academic literature has claimed methodological and technical shortcomings with the Formula [7]. Several studies argue that although age is the dominant factor in determining the level of allocations, deprivation, comorbidities and functional impairments are stronger predictors of healthcare spending than age, highlighting the limitations of using age alone as a primary driver of healthcare need or healthcare inequality [29, 48, 53, 122]. Studies also report “considerable differences” between formula’s allocations and the actual funding received, leading to under- and over-funding in different regions as well as political reluctance to adjust allocations in ways that could address age or deprivation related inequities ([29] p.10–12, [7]).

#### Age in commissioning and ethical frameworks

The Department of Health’s 2009 report on achieving age equality in health and social care highlighted concerns about whether commissioning serves the needs of all age groups equitably ([13], p.32). The report recommended “to include an age dimension in local audit and planning processes with clear action to advance age equality and tackle discrimination” (p.6). Later, the Government Equalities Office guidance (2012) stressed that commissioners “should provide appropriate services for individuals with similar needs which may include age-related needs” ([45], p.8).

Most commissioning bodies at both national and regional levels have established ethical frameworks to guide their resource allocation decisions. At the national level, NHS England (then known as the NHS Commissioning Board) has its own framework for decision-making [86] which, whilst interim, guided decision-making throughout NHS England’s existence. The Framework does not mention age as a priority criterion but embeds it within the NHS’ equality and non-discrimination commitments. At the regional level, many Integrated Care Boards (ICBs) and Priorities Forums have also set out their own frameworks. Despite some differences between frameworks, most guidelines emphasise that decisions should be guided by clinical need. Several frameworks explicitly state that committees will not discriminate on the basis of age (among other characteristics) and individuals should not be unjustifiably advantaged or disadvantaged because of their age. However, these frameworks also make clear that age (and other characteristics) can be taken into account where they may affect need, capacity to benefit, and clinical effectiveness. These include frameworks drafted by: NHS Priorities Forum [Bedfordshire, Hertfordshire, Luton and Milton Keynes] [93]; NHS and North East London Health care Consulting [91]; NHS Nottingham and Nottinghamshire Commissioning Senior Leadership Team [92]; West Yorkshire Health and Care Partnership & NHS [141]; Hampshire, Southampton, Isle of Wight ICB and Portsmouth Priorities Committee [50]; Kent and Medway ICB [61]; and Together for Devon [135]. Others, such as the South East Region Policy Recommendation Committee’s Ethical Framework 2024 emphasise that NHS care should never be restricted on the basis of a protected characteristic but do not specifically mention age [85, 90].

#### Age in clinical guidance and decision-making

Through the years, the National Institute for Health and Care Excellence (NICE) has addressed the role of age in their decision-making through equality-based frameworks. Earlier guidance, such as their 2008 social value judgements document warned against denying or restricting care simply on the basis of age but allowed age to being taken into account when it is a reliable indicator of risk, response to likely treatment, or when no suitable alternative marker is available. This has remained part of their methods and process manuals to the present time [96, 106, 107].

In more recent NICE guidance age is less frequently presented as a stand-alone criterion in decision-making. In the NICE Principles, age features as one of several protected characteristics under the Equality Act 2010, and through NICE’s broader statutory duty in relation to non-discrimination and the reduction of health inequalities set out in the Health and Social Care Act 2012 (Equality Act, 2010; Health and Social Care Act, 2012). The Principles also advise that there should be clear justification for targeting specific groups, such as subgroup evidence, a statutory mandate, or a clear equity-based rationale [107].

In methodological guidance, age is included among social characteristics for which explicit use is restricted. The NICE health technology evaluation manual (published 2022; updated 2025), which introduced new provisions for assessing health inequalities, states that subgroup analyses “based solely on social characteristics” should be excluded from cost-effectiveness analyses ([106], p.97). However, recent appraisals, such as for the treatment for Hodgkinlymphoma [109] and allergic rhinitis [108] demonstrated that even when not explicitly considered, age may still influence both the evaluation of a treatment’s value and patient access. Questions remain about the ability of current cost-effectiveness models, which often average results across age groups, to adequately capture inequalities in outcomes between younger and older patients. Notably, in measuring health-related quality-of-life, NICE assessments treat children and young people separately, with age-appropriate utility measures ([106], p.72).

Age nonetheless is a decision-making factor in specific guidance. For instance, in the In Vivo Fertilisation (IVF) guidelines, age-based eligibility criteria are set for women under 42 [103]. Although a surveillance review in 2015 found no equality issues with these age limits [97], concerns were raised. Clinical Commissioning Groups (CCGs) were applying the guidance inconsistently with some CCGs introducing stricter criteria or placing upper age thresholds for male partners [59].

Distinct healthcare policies across the devolved governments further complicate the role of age in decision-making. New treatments in Scotland are appraised independently of NICE [24]. In England different age bands are used to those in Scotland and Wales, both for national cancer screening programmes and for the treatment of certain conditions, including neurodegenerative disorders [88, 104, 127, 137]. Although these differences have not been found to constitute age discrimination but rather the distinct procedures and priorities of each system, some scholars have proposed alternative approaches as fairer to those based on fixed age bands. These include the use of risk stratified protocols to improve sensitivity in screening, though their application remains contested [123].

#### Age in patient prioritisation

In patient prioritisation Horton [55] observes that age is sometimes used as a proxy “when more precise markers for benefit, harm, or patient interest are unavailable” (p. 92,97), or “when other indicators carry ethical risks, such as generating stigma” (p. 95). This practice, however, has led several scholars to caution that age should not be confused with the impact or presence of other health conditions (such as comorbidities) when deciding who is eligible for treatment [75]. Others note that prioritisation decisions should consider a patient’s functional status and individual needs rather than age alone [74].

#### Age in UK COVID‑19 policy

During the COVID‑19 pandemic, age was frequently referenced in national policies and guidance but its role varied. In the vaccine rollout prioritisation strategy, age was used as a key risk factor with the programme starting with care home residents and people over 80 before extending to clinically vulnerable individuals over65 [62]. Age also influenced digital inclusion strategies aimed at maintaining healthcare access during lockdowns. In Wales £3 million was invested in digital devices and connectivity, with particular focus on older adults in care homes, to reduce isolation and support healthcare access [10]. By contrast, England and Northern Ireland directed most of their digital access funding toward education with little evidence of equivalent support for older adults’ healthcare needs [2, 73].

Age also featured in clinical guidance but its role was contested. The UK Government classified everyone over 70 as “clinically vulnerable” but such blanket classification was criticised by several scholars and policy groups for oversimplifying risk and overlooking the diversity of health status among older adults [4, 124, 134]. In NICE’s rapid guidance on critical care, explicit age thresholds were not set but clinicians were advised to use the Clinical Frailty Scale (CFS) to guide individualised decisions about access to intensive care [74]. Similarly, local ethical frameworks such as the Devon Ethical Framework for allocation and withdrawal of organ support and/or treatments during the COVID-19 pandemic explicitly rejected age as a categorical factor in treatment decisions [135]. These decisions also aligned with statements by several scholars and policy organisations arguing that the use of ‘age cut-off’ practices, even under severe resource constraints, can be potentially unlawful [11, 69, 129].

### Children and young adults in policy and healthcare decision-making

#### Children in healthcare policy, planning and funding

Although the Equality Act 2010’s protections against age discrimination formally apply only to those over 18, to avoid weakening existing legal protections for children [45], the Government Equalities Office and Equality and Human Rights Commission guidance (2015) clarifies that the Public Sector Equality Duty (PSED) requires public bodies to “consider all individuals when carrying out their day-to-day work” which includes children and young people [47]. The NHS practice guide for achieving age equality in health and social care clarifies that like adults, children, can also face forms of disadvantage and differential treatment in healthcare, some of which may raise concerns about direct discrimination, often rooted in stereotyping, and indirect discrimination caused by structural inequalities such as homelessness [94]. It also stresses that age-sensitive care is not discriminatory if it is objectively justified, and calls for “a child-centred, outcome-led vision… joint planning and commissioning, pooled budgets… and integrated front-line delivery organised around the child” (ibid, p. 238).

However, despite policy emphasising early-years investment and evidence suggesting that investing in children’s care is highly cost-effective and associated with improved outcomes in adulthood [40, 125], paediatrics remains underfunded with profound implications for children’s health outcomes and long-term population health [80, 120]. Since the 2012 Health and Social Care Act, decentralised commissioning has resulted in a “persistent mismatch between demand and commissioned supply” (The Strategy Unit 2021). Different commissioning committees set their own priorities [17] and have their own funding arrangements [66]. These choices often lead to uneven care provision and widen the gap between child and adult services [1, 3, 63, 80].

Evidence also shows that workforce planning and performance targets continue to be framed around adult care [42, 133] leaving children’s services deprioritised in policy attention [28, 125]. Policy organisations and scholars note the absence of clear policy mandate to address the distinct, and diverse social, emotional and cognitive needs in early years of life as a “core business” across the NHS ([125], p.73, [80]). Problems with safety and consistency continue to be reported across the system, from preventive measures to specialised treatment. Examples include the potential benefits of early life intervention to address adult cardiovascular health [12] and variation in dental sedation practices [140].

In mental health, concerns have been raised at both policy and implementation levels. The 2018 ‘Green Paper on children and young people’s mental health’, a government policy consultation document, was criticised for having a lack of commitment to address the higher level of need in particular groups, including looked-after children [28]. Critics pointed to underinvestment in deprived areas [28, 42] and the lack of recommended interventions for the most socially disadvantaged children, including those in the criminal system, despite clear evidence of their heightened needs ([28], §63). Discrepancies between clinical guidance and practice are also reported. Evidence shows that most mental health services breach NICE timeliness standards [80]. In some areas children are placed in adult psychiatric wards in breach of the Mental Health Act code of practice [42]. Long delays [125] and complex referral routes combined with “little agency or consistent advocacy” for children ([39], p. 2) are consistently reported.

Concerns regarding children’s care were particularly pronounced during the COVID-19 pandemic. In the recovery phase, investment focused heavily on adult backlogs pushing children’s services further down the agenda. Professional bodies have warned that children are being treated as “second-class citizens” in a system designed around adult needs and have called for dedicated funding for children’s health and for Integrated Care Systems to align their priorities more closely with the needs of the paediatric population [120].

#### Transitional care in policy and decision-making

The NICE quality standards on transition from children’s to adults’ services call for care for people aged 0–25 that is better integrated and designed to meet their developmental needs [104, 125]. Yet evidence shows that decision-making processes for transitioning are typically based on fixed ages, between 16 and 18 years, rather than a young person’s developmental readiness [37, 114] and often without a clear plan or documentation [136]. As a result, many children and young people experience interruptions in treatment, gaps in follow-up care and have little involvement in their transition planning [63, 80]. These challenges are well established in young people with mental health conditions, juvenile idiopathic arthritis or kidney disease (Abbott et al. 2019; [3, 63]).

Resources to support young people during transition are also limited [66]. Only 68.2% of acute NHS trusts have adolescent services, only 24.5% provide adolescent wards, and 32.3% run adolescent clinics [80, 117]. Reports consistently point to poor planning, under-resourcing, and services that fail to meet this group’s long-term developmental needs [3, 17, 39, 63, 110].

Inequitable access to adult services and suboptimal care [74, 75, 80] combined with major life changes in young people’s lives (such as leaving school, entering the workforce, or gaining social independence) increase the risks of health deterioration [17, 39]. These factors may lead to poorer health outcomes ([1, 63], p. 6) and in some cases irreversible harm [64, 120]. Young people with overlapping vulnerabilities, for instance those who are homeless or in foster care, are often missed by current services [5, 28, 125].

These problems are especially pronounced when young people transition from Child and Adolescent Mental Health Services (CAMHS) to Adult Mental Health Services (AMHS). The lack of national and local policy to guide integrated commissioning for transitional mental health care as well as inflexible and poorly connected mental health care services [126] often leave this group without adequate support (The Strategy Unit 2021; [39, 63]). Moreover, while CAMHS generally provide holistic care based on a child’s developmental needs, AMHS are not designed to meet young people’s needs as they tend to focus on crises and to follow rigid diagnostic criteria [39, 63]. Young people with neurodevelopmental conditions such as ADHD or autism are particularly affected [16, 39, 80].

Young people, aged 18–25 years, with life-limiting conditions are reported among those most impacted by rigid age thresholds in service provision. Despite the NICE quality standards on transition, standardised guidelines are still lacking [37, 63] and evidence on effective transitional care for this group remains limited [3]. This group is reported to often receive disrupted and inappropriate care, poorer access and higher re-referral rates compared to their younger peers and poor healthcare outcomes [80, 110, 136].

### Working-age adults in policy and decision-making

#### Working-age adults within policy and service organisation

Working age adults are the largest group within the population but their needs are not clearly defined in healthcare policy. Clinical guidance and services often position them within a broad ‘working-age’ category, as a homogeneous group ([37], p. 25). This approach has been considered to overlook variation in need and the presence of complex and intersecting health factors, including age, disability, ethnicity, socioeconomic position [12, 37, 67, 76]. Studies have shown that limited recognition of the varied needs of this group results in inconsistent clinical decision-making and low clinician awareness of unmet needs disproportionately affecting those from ethnic minorities, as well older or socioeconomically-deprived people [12, 70, 130].

Evidence also shows that the needs of older working-age adults with complex needs, intellectual disabilities, or organic mental illness are systematically unmet as they often fall between the remit of working adult and older people’s services [113]. Adults with autism have been identified as a group for whom services remain underdeveloped. National policy, including the 2019 NHS Long Term Plan, have been criticised for limited attention to timely diagnostic pathways, as “autism has long been viewed as a paediatric condition” ([113], p. 1).

Concerns regarding the lack of policy attention to the varied needs of working age adults have also been reported in preventive care. Having endorsed the World Health Oraganisation (WHO)’s Immunisation Agenda 2030, the NHS is committed to deliver vaccination across the life course and to target under immunised groups of any age. However, unlike childhood vaccination programmes, adult vaccination efforts lack established commissioning and delivery systems and have limited coverage and outreach [9, 14, 31]. Lack of formal planning and consistent processes tend to affect socially disadvantaged groups the most [14].

#### Gender inequalities in policy, funding and clinical decision-making for working-age adults

Recently women’s health has received increased attention in national policy [12, 128]. The inaugural Women’s Health Strategy for England in 2022 highlighted a number of women’s health services relevant to women aged 40–65 including sexual health and wellbeing services, contraception and gynaecology services including menopause care [128]. Improved public awareness and acceptance through online information and social media campaigns have also helped improve access and uptake of menopausal care options and hormone therapy [67]. However, evidence shows that due to recent governance changes and long-standing practices within the NHS that have repeatedly raised concerns about discrimination, women’s care continues to face significant shortcomings [32].

Since the introduction of the 2012 Health and Social Care Act (HSCA), commissioning for sexual and reproductive health (SRH) services has moved to local authorities with the aim to improve responsiveness to local needs. This change, however, also led to fragmented services and postcode-lottery of care [76]. Women in perimenopause and those accessing the services for the first time are particularly affected [72]. Barriers to accessing long-acting reversible contraceptives (LARCs) are mainly reported in those older in the working age- group and those living in certain areas; studies show that these factors may be contributing to the rise of abortion rates in recent years [76]. Women living in deprivation also have a higher risk of infertility but evidence shows that they face greater barriers to treatment [27].

Significant inequalities are reported in In Vitro Fertilisation (IVF) treatment because of uneven funding constrained by age limits, regional restrictions and lack of health practitioners’ education to better support women according to their life stage [72, 76]. Those older, obese or from ethnic minorities are less likely to be referred to urinary incontinence (UI) surgical care, and to receive surgery, despite evidence of safety and higher prevalence of UI in older age [43]. Likewise, misconceptions about cardiovascular disease (CVD), heart failure, stroke and arrhythmia, which have been traditionally thought as older adults’ diseases, have significantly affected diagnosis and treatment, particularly for those younger in this group [12]. Unmet health needs are also reported for working-aged men, especially in preventative services due to fragmented commissioning and workforce shortages. Those from migrant communities and socioeconomically disadvantaged groups are especially impacted [14]. In mental health care, factors such as cultural stigma and help-seeking behaviour clearly contribute to healthcare disparities [21], but the absence of dedicated health strategies for the distinct needs of this group is also notable. For instance, in suicide prevention policy it is well acknowledged that men account for around three-quarters of all suicides but their needs remain only indirectly addressed through broader programmes such as the NHS Mental Health Implementation Plan, without specific attention to their increased risks [83].

### Older adults in policy and healthcare decision-making

#### Older adults in policy, funding and system design

Concerns about differential treatment of older adults have long been highlighted in UK health and social care policy. The Departments of Health’s 2009 policy and guidance report on age equality warned that “narrow assumptions about older people” can limit their access to services and that younger individuals with comparable levels of need are more likely to receive comprehensive support packages ([13], p. 7, 37–38). The government later confirmed that the purpose of implementing the age discrimination ban without exemptions, under the Equality Act 2010, was to tackle disparities in both funding and service delivery and to provide to those affected a clear route to challenge treatment that may constitute unlawful discrimination ([45], p. 9). Subsequent NHS guidance emphasised that neither a “one size fits all ages” approach nor the “use of age as a way to limit access to services without any evidence” are acceptable ([94], p. 1). It also calls for integrated and age-sensitive service design and the active involvement of older adults in commissioning and monitoring processes [101].

Alongside these policy commitments, improving outcomes in later life has become a priority of the UK Government [111, 112]. In 2019, Public Health England (PHE) and the Centre for Ageing Better issued a consensus statement on ‘healthy ageing’ aiming to make England “the best place in the world to grow old” and a commitment to narrow the gap between the richest and poorest [111]. Proposals in the government’s 2022 white paper “Integration and innovation: working together to improve health and social care for all” called for better integration both within the NHS services and with social care services and other authorities to support earlier intervention and more consistent access to services [23, 131].

Despite these high-level policy commitments, evidence shows persistent differences in how healthcare priorities are set for older people [121]. While barriers to equitable access have been linked with relocation of older adults to underserved coastal and semi-rural zones [142], public health budgets have increasingly prioritised early-years interventions [26, 29] with ‘invest to save’ elements largely absent for interventions targeting older individuals ([54], p. 2, [7]). Moreover, devolved policy making and variations in drug approval processes, combined with nation-specific targeted initiatives (such as Wales’ Older People’s Strategy) have resulted in variation in prescription access across England, Scotland, and Wales and uneven service entitlements across the UK [10, 45]. Policy organisations and scholars have raised concerns for the economic evaluation tools used in resource allocation particularly in conditions like Alzheimer’s disease and revascularisation [36]. The NHS resource allocation formula has been criticised for perceived methodological and technical shortcomings that fail to adequately reflect real-world factors, such as comorbidities ([15], p. 61) and for favouring treatments for diseases more common in younger groups ([54], p. 2). Some commentators suggest a broader ethical shift in the NHS toward “equalising health outcomes” rather than “equalising access” based on need, may have unintended consequences for older groups [7].

In service design, evidence shows that the changing and heterogeneous needs of this group have received little attention [68, 71]. Health inequalities are reported to be particularly pronounced in mental health as services have historically been organised around the division between working- aged and older-aged adult populations [44]. Responses to mental health for older adults have largely centred on dementia, leaving other conditions, such as depression, anxiety, and substance misuse underdiagnosed, despite strong evidence of effective treatment similar or even better to younger adults [78]. Research suggests that weak data, limited guidance, and a lack of leadership contribute to the persistence of these problems [77–79] reflecting challenges in translating policy ambition for improving outcomes in later life into consistent implementation across the spectrum of older people’s health needs. Reports from the National Development Team for Inclusion (NDTi) highlight a lack of joint understanding about what “age equality” means in practice, as well as a lack of monitoring of age-related inequalities in healthcare access and outcomes emphasising that progress depends on broader cultural and structural change supported by positive attitudes to ageing and age-aware commissioning [77, 79].

Scholars and policy organisations note that inequalities in access affecting older adults reflect a broader pattern across UK and demonstrate a health system which is underprepared at times giving rise to practices that raise concerns about age discrimination [15, 121]. Studies indicate that older people have fewer treatment options and GP consultations, more prescriptions ([41], p.7) and poorer healthcare outcomes compared to younger groups [8, 13, 15, 30, 45, 65, 116], with only partial exceptions (e.g. the fast track TIA clinics in stroke care [119]. Vulnerable people within this group face accelerated age-related decline, multimorbidity and social disadvantage and are thus disproportionally affected [60, 67]. Evidence also shows that as services remain focused on recovery rather than supportive care, many people in old age continue to die in hospitals rather than palliative care settings [5].

#### Older adults in clinical guidance and clinical judgment

NICE guidance pays particular attention to patient-centred approaches in older people’s care and their active involvement in decisions about their treatment. Its recommendations for social care and multimorbidity [99, 102] call for better system navigation, better coordination between health and social care, frailty assessment, and more personalised plans [102]. Complimentary guidelines on transitions from hospital to community care [100] and older people’s wellbeing [101], focus on the importance of individualised assessment, access to effective care management and specialist geriatric units. They also draw attention to the role of programmes that help older adults stay independent and maintain mental wellbeing.

However, implementation remains restricted largely due to limited data and the continued underrepresentation of older adults in clinical research. NHS England’s “improving care for older people”, and broader research evidence [37, 49, 81], underscore the need for better ways for understanding and measuring key stages of frailty, which remain loosely defined and under-measured in practice. This lack of evidence leaves gaps on how best to use preventive medicine for frail populations and how to estimate life expectancy in treatment decisions [99, 102]. Further evidence gaps include a lack of sufficient holistic and community-based assessments, decisions on deprescribing, and limited UK-based evaluation evidence on surgical outcomes [75]. Limited evidence is also noted on interventions targeting mid-life risk factors that may influence later-life independence or mental wellbeing [98] and for the cost-effectiveness of geriatric versus general ward care and post-discharge outcomes [101, 102].

Gaps in clinical guidance affect clinical decision-making. Research shows that clinicians often use age informally, as a proxy for factors such as frailty, patient preference and capacity to benefit from treatment when deciding who gets referred, which treatments to offer, and how information should be shared, often to the disadvantage of older patients [5, 71, 81, 116]. Studies across surgery, cardiology and cancer care show that clinicians do not consistently follow NICE guidelines when treating older patients and that their decisions may be affected by implicit biases, which in some cases may contribute to patterns that raise concerns about indirect discrimination [8, 115, 132]. Despite evidence that older people can benefit from these services, referral treatment and rehabilitation rates remain low ([132], p.6) reflecting healthcare professionals’ assumptions regarding patients’ preferences and needs rather than patients’ failure to seek help ([75], p.3, [8, 81], p.259). In cancer care older people are less likely to receive interventions, often receive diagnosis at late stage or through emergency routes and have poor survival rates that neither comorbidities nor patient choice fully account for [52, 75].

Although frailty and pre-morbidity functional status are stronger mortality predictors than chronological age ([129], p.60) evidence shows age-related assumptions associated with lower referral rates in palliative care [15, 65, 75]. Greater reliance on subjective clinical judgment has also been linked to increased inequalities in access to intensive care services [15, 30]. Many frail older adults lack formal diagnoses and are reported to often experience difficulties in accessing community-based end-of-life services with increasing numbers deteriorating or dying of frailty without necessary support ([116], p.757).

In mental health care, studies show that access criteria are often tied to age or cognitive status and are associated with restricted diagnosis and treatment for those over 65 [37, 118]. These practices have been linked to assumptions about reduced treatment benefit from psychological therapies in later life [79]. These assumptions entrench long-standing inequalities in healthcare access, which can give rise to inequities and raise serious concerns about discrimination against older adults.

### Policy and decision-making for populations with age-atypical profiles

Populations whose age profiles fall outside the statistical norm, such as adults with early-onset frailty or rare diseases, and children with life-limiting conditions, are acknowledged as distinct groups with specific care needs [33, 57, 113]. Several challenges, such as limited local capacity and constraints in funding that affect the stability of specialised services are acknowledged in key documents including the Commissioning Guidance for Specialist Palliative Care [6]; NHS England’s guidance note on ‘GP Practices Serving Atypical Populations’ [89], two reviews of specialised commissioning [33, 58]; as well as in policy on rare diseases, including the England Rare Diseases Action Plan [24] and the UK Rare Diseases Framework [22].

Studies exploring patients’ and their families’ experiences highlight inequities in healthcare access that have received less attention in these policy documents and guidelines. These include postcode variation in access to services, long travel distances for specialist care, and age-inappropriate equipment [57, 68, 110, 136]. To address these gaps scholars have proposed a shift from generic age-based provision to a needs-based model to better meet the needs of numerically smaller groups with age atypical profiles and have highlighted the need to better integrate specialist support within mainstream care [3, 37].

## Discussion

Advancing equality is a core value in the NHS Constitution, which commits the NHS to respecting equality and human rights and working to reduce inequalities in care access, experience and outcomes [147]. Reflecting this, NHS England [88] notes that people who share protected characteristics are more likely to face such inequalities and that, in some cases, decisions which advance equality of opportunity may also help reduce health inequalities, and vice versa.

Our findings identified persistent age-related inequalities in access to and provision of care that remain insufficiently addressed in current healthcare policy. Although age and age group considerations were not absent from decision-making frameworks, limited and inconsistent attention was given to how age is operationalised in practice. This lack of clarity raises concerns not only about health outcomes, but also about the ways in which age equality duties are understood and applied in healthcare decision-making, and across different policy levels.

The review identified four distinct ways in which age features in healthcare policy and decision-making, each of which affects access to care through different mechanisms and with different implications across the life course (a) age used explicitly as an eligibility criterion, (b) age being used as a proxy or heuristic, (c) age explicitly prohibited from being used and (d) age not explicitly considered yet functioned as an invisible or omitted factor.

First, when age is used as an explicit criterion of access, it is usually to organise services, for instance the division between CAMHS and AMHS, so that care provided can better accommodate different population needs ([24]; NHS England 2020). Age may also serve as a formal eligibility criterion for specific interventions, such as in IVF or cancer screening programmes, where evidence supports age-based distinctions for safety or effectiveness reasons, or where age is deemed a more eligible criterion than alternatives [25, 55, 103]. In these cases, age directly determines who has access to care and under what conditions. Although such differentiation does not in itself constitute direct discrimination, provided it can be justified as a proportionate means of achieving a legitimate aim, our review identified several ethical concerns with the explicit use of age and in particular with how age-based criteria work in practice. Chronological age is an imperfect indicator of health need and thus does not reflect the heterogeneity within age groups. Age thresholds were found to often overlook developmental differences in children, particularly during transitions to adult services, and to obscure variation in care needs and functional ability among adults [34, 63, 78, 110]. Individuals with age atypical conditions, as in the case of adults with early-onset frailty or children with life limiting conditions are particularly affected [113, 136, 171]. Rigid age boundaries were in some cases reported to serve as barriers to clinically appropriate care, especially where local services do not have the capacity to offer age-sensitive pathways, as in the case of adolescents treated in adult mental health services [42]. Equality concerns were particularly raised where age thresholds differ between commissioning bodies (for instance in IVF treatment), especially where inequalities in access were due to local financial constraints or different priorities, rather than clinical evidence [59].

These issues are directly relevant to questions of necessity and proportionality. Decision-makers must consider whether less restrictive alternatives exist and how the administrative benefits of age-based criteria are weighed against their exclusionary effects. These issues also highlight fundamental ethical tensions. On one hand, the use of explicit age-based differentiation may allow for greater targeting of needs and resources but on the other there is risk that such differentiation may restrict access to care solely on the basis of age, thereby excluding individuals who could otherwise benefit from the same care on equal terms.. Importantly, our findings showed that age-related inequalities associated with explicit age criteria are not isolated implementation issues but reflect a broader fragmentation between policy aspiration and practice. Although the NHS endorses the “life-course” approach [84, 95, 162] and patient-centred approaches to care [102], services continue to operate within rigid age boundaries that often fail to meet the needs of those at the edges [79, 80].

Secondly, the review found that age is often used in decision-making as a proxy for needs, risks, or potential benefits [55, 96, 107]. Sometimes using age in this way was made explicit. For instance, in the Weighted Capitation Formula where age serves as a proxy for population healthcare needs, increasing healthcare funding for areas with older populations [87]. While we did not find concerns regarding potentially discriminatory practice raised in principle with this approach, scholars and policy groups have discussed methodological limitations [7] and inconsistencies with actual funding allocations [29]. These inconsistencies may contribute to or exacerbate health inequalities, and in some contexts inequities, affecting older populations, particularly in deprived areas. In terms of proportionality these discrepancies raise questions about whether age functions as an adequate proxy for need, and whether reliance on age alone can be justified compared to more precise alternatives.

It also raises ethical concerns especially in cases where age remains a useful metric even when more precise indicators of need, such as frailty or deprivation, are taken into account. In such cases, decision-makers may face an important epistemic and distributive challenge on whether and if so, when, it is acceptable to characterise people based on a trait that is statistically useful but socially sensitive. From a legal perspective such use may still be justified if it contributes to a legitimate aim and where it can be shown that reliance on age is proportionate compared to available alternatives. Yet, from an ethical perspective decision-makers should also consider whether the marginal predictive value of age is sufficient to justify differential treatment and how the benefits of more accuracy should be weighed against the risk of reinforcing age-based stigma. Balancing these considerations is a complex task Ahat the proportionality test despite providing a legal standard does not resolve.

We also found that age was often used, implicitly, as a proxy in clinical practice, affecting healthcare access through what is assumed in routine decision-making. Clinicians often rely on age when more precise markers for need such as benefit or frailty are not available [55, 115]. But such implicit use of age has also been found to contradict clinical guidance and can lead to inappropriate treatment or exclusion from treatment, despite evidence of benefit. Such practices raise serious questions about how age-equality requirements are actually operationalised, and in some instances may even constitute indirect discrimination [15, 116]. Although these issues have been well-documented in cases such as geriatric surgery [115] and reproductive care for older working women [43], their informal and routine nature means they are difficult to identify and scrutinise in practice.

The third way in which we saw age feature in policies and frameworks, is when its use is explicitly prohibited. For instance, where NICE bans the use of age categorisation in subgroup analysis on the basis of differential cost-effectiveness (NICE, Methods Manual, 2025b) on the rationale that social characteristics should not influence decisions about cost-effectiveness [96]. Such prohibitions are clearly intended to prevent direct discrimination. However, the exclusion of age from analysis may limit the ability to identify age-related inequalities and inequities particularly where differences in outcomes or needs are systematically associated with age. This may result in age continuing to influence decisions indirectly by what is left unmeasured (such as comorbidities common in older adults) or through the reliance on average outcomes based on typical age profiles [15, 36]. Critics have pointed to how such invisibility may result in unexamined age biases persisting in resource allocation decisions and reinforcing healthcare inequities that formal policy does not intend to justify [36, 54].

Finally, age is often mentioned in commissioning frameworks amongst the protected characteristics and amongst the categories in which sub-group differentiation might be permitted in order to target groups with poorer health access or outcomes [87]. However, we found little explicit guidance on how age-related considerations should be incorporated into decision-making and on how inequities should in practice be addressed. This lack of operational guidance creates a gap between equality commitments and implementation, and leaves decision-makers to navigate their way through the ethical tensions we identify above without sufficient ethical and legal support. Age may still feature implicitly, in a way that is more likely to be biased and inaccurate, allowing outcomes across conditions and services to be influenced indirectly without a central mechanism to ensure consistent interpretation and application of age equality duties.

Our results showed that these fundamental tensions were often resolved in one of two ways. There was either a continued reliance on rigid age-based criteria—which risk overlooking diversity within age groups—or the adoption of an “age-blind” approach—which can fail to recognise age-related variation in need and responsiveness to care, further inviting informal and inconsistent uses of age in decision-making. National policies and reviews have warned against both extremes [13, 45] for failing to meet age equality requirements, but our findings indicate that these practices remain widespread in the way care is provided.

This discrepancy may reflect limitations in current guidance, which offers little practical direction on how the legal requirement that age differentiation must be “objectively justified” should be operationalised and on how decision-makers can meet their equality-based duties [77, 79]. Although it would be unrealistic for guidance to capture all the ways in which age might feature in decision-making, and decision-makers in many cases will be expected to use their own judgement [105], the absence of clear direction may potentially disincentivise active and explicit use of age in decision-making, where it could be relevant to meeting age-equality requirements. This may be particularly concerning if decisions are being made based on past practice [153], especially where past practice predates advances in equality law. Moreover, as the law has yet to be tested in the court in this context [55], decision-makers may be reluctant to move away from past practices which seem to have faced little judicial challenge. Such omissions may therefore result in greater reliance on implicit or informal uses of age, which are less visible and more difficult to evaluate in terms of inequity, or potential discrimination.

Our aim, however, is not to suggest a change to the law, nor do we want or expect significant substantive limitations on when, where, and how age can and should be used in these cases. It is our opinion that these issues are too fundamentally complex and contextual for any detailed specification of the law or in guidance to be accurate or appropriate. The proportionality test is legally appropriate, especially in so far as it sets out a broadly procedural approach to determining these fundamental issues of justice. Such proceduralist approaches are widely used in contexts of complex ethical tensions, where individuals are likely to disagree on how to resolve the tensions, including in healthcare justice and resource allocation cases [18, 19, 82]. However, procedural approaches should not be considered any less demanding; for the outcome of a procedure to be just and legitimate certain conditions need to be met and standards upheld [56].

Our primary ethical concern then is that the literature we encountered not only highlighted fundamental ethical tensions in age-related equality, but also seemed to show that these tensions have not been fully recognised, considered openly and rigorously, and that there is little ethical guidance and legal precedent to ensure robust procedural standards are maintained. Discrepancies in approaches thus seem to demonstrate not a degree of appropriate subsidiarity or responsiveness to different ethical factors in distinct context, but a deeper dereliction of duty where decision-makers have presumed either an explicitly age-differentiated or “age-blind” approach is justified and applied it with little further consideration.

Another important finding of this review was that despite references to age discrimination within policy documents, there was limited attention paid to the intersection between age and other forms of social inequality and their implications for equitable access to healthcare for patients at different life stages. Our findings indicated significant inequities affecting working-aged women living in deprivation, looked after children and older adults facing stigmatisation [27, 28, 116] which remain unrecognised and unaddressed. Moreover, most of the relevant evidence on healthcare inequalities across the life course came from the academic literature rather than policy sources. Notably, ‘working age adults’ despite being the largest population group were the least discussed in the reviewed documents.

Another particularly surprising result of our findings was the relative lack of academic literature we encountered on dementia, despite general acknowledgement that people living with dementia may be particularly vulnerable to indirect discrimination, as universal policies and rules may disadvantage them by failing to take into account their special needs [159]. It is tempting to speculate that this absence may indicate that dementia—and its effect on service eligibility, access, and resource allocation—is not perceived as an issue of age-related equality or justice, but either as its own distinct issue of justice or not an issue of justice at all. However, either conclusion cannot directly be supported by the findings of this review, though this abductive interpretation may justify further exploration into this inference. We also found little evidence on systematic evaluation tools and mechanisms to identify compounded inequalities and inequities affecting specific groups across the life course even where inequitable care access is known to exist. This may further suggest that age equality is not yet consistently embedded in healthcare policy and decision-making.

Our findings thus point to issues that require significant policy attention. Of particular note, are illustrative examples including issues faced by frail older adults dying without palliative support, vulnerable children with mental health conditions falling through service gaps, working-aged men at high suicidal risk being underserved and working-age ethnic minority women with unmet psychological and reproductive health needs [3, 5, 12, 14, 27, 116]. However, these cases, whilst visible and well documented, are unlikely to be exhaustive of age-related inequalities and inequities which may persist, even in the absence of explicit discriminatory intent. The need to focus on strengthening decision-making processes and guidance to support consistent and appropriate consideration of age, alongside systematic evidence gathering and monitoring is therefore paramount. Future research should examine barriers to the implementation of age equality and how decision-makers across the healthcare system interpret and apply their age equality duties. Future research should also explore how these interpretations may affect healthcare access and outcomes particularly for groups with overlapping vulnerabilities not covered in this review.

The ethical tensions identified in this review connect to broader debates in distributive justice and priority-setting [20, 38, 51]. Our aim in this review is not to adjudicate between different ethical positions or to advance a particular theory of justice. Rather, what this analysis has shown is that, regardless of the ethical framework adopted (e.g. sufficietarian or prioritarian), the current use of age in policy and practice often lacks the explicit ethical reasoning required to justify differential treatment.

## Limitations

This work has several limitations. Our aim was to provide a comprehensive picture of the ways in which age and age-groups feature in policies and healthcare processes that determine healthcare provision. While a wide body of policy, academic and grey literature was synthesised, it is possible that some practices may be under-represented due to unpublished documentation. Although the review focused on the UK health system, most of the available material concerned England. Moreover, due to a lack of age-specific data in official documents and limited attention given to age-related inequalities in policy and research [169], some important age-based healthcare inequalities and inequities may not have been captured in this review. Finally, the study does not undertake a full proportionality analysis of individual policies or practices. This would require detailed case-specific evaluation beyond the scope of a scoping review.

Despite these limitations, the review brings needed focus to an important issue which has received little attention so far in healthcare policy and practice and identifies key areas for further research and policy action. We hope that this work will help support efforts to strengthen age equality across the healthcare system.

## Conclusion

Two interrelated findings emerged from this review. First, persistent age-related inequalities, some of which constitute inequities and in certain contexts may raise concerns about discrimination, remain insufficiently recognised in current healthcare policy. Second, there are limited ways in which age is addressed as an equality concern in healthcare policy and decision-making frameworks. Both these findings underline an ethical concern: significant procedural failings in healthcare decision-making undermine the justification and legitimacy of age-related policies and practices and threaten age-equity. There is a clear need for systematic evidence gathering and monitoring to identify and address age-related inequities, as well as lack of practical guidance to support decision-makers to meet their equality-based duties in practice. The routine use of age-sensitive equality impact assessments across services could serve as an important first step in supporting more systematic identification and documentation of currently overlooked health inequalities across the life course, and among age-groups, strengthening accountability and transparency in decision-making and in informing future research and policy development.

## Supplementary Information

Supplementary Material 1.

## Data Availability

The dataset supporting the conclusions of this article is available in the protocols.io repository: (https://dx.doi.org/10.17504/protocols.io.36wgqp4w5vk5/v1).

## Abbreviations

AMHS: Adult Mental Health Services:
CAMHS: Child and Adolescent Mental Health Services
CFS: Clinical Frailty Scale
CCGs: Clinical Commissioning Groups
DHSC: Department of Health and Social Care
QALYs: Quality Adjusted Life Years
HSCA: Health and Social Care Act
NHS: National Health Service
NICE: National Institute for Health and Care Excellence
ICBs: Integrated Care Boards
IVF: In Vitro Fertilisation
LARCs: Long-acting Reversible Contraceptives
SRH: Sexual and Reproductive Health
WHO: World Health Organisation
