# Supplementary material for: Age equality in UK healthcare policy and decision-making: a scoping review
Source: BMC Med Ethics. 2026 Jun 24;27:189. doi: 10.1186/s12910-026-01508-1 (PMC13551792; doi:10.1186/s12910-026-01508-1)
Supplement: Supplementary file 1 [file 12910_2026_1508_MOESM1_ESM.docx]

# Appendix 1- PRISMA

Studies from database searching **(n = 1260)**

References from manual searches **(n =80)**

[Additional relevant articles matching review criteria identified from references of eligible articles and manual searches]

**Identification**

Studies assessed for eligibility (full-text) **(n = 242)**

Studies screened by title and abstract **(n = 826)**

Studies excluded **(n = 104)**

Full-text not available open access (n=1)

Normative content not evidence (n = 11)

Useful Background (n = 17)

Withdrawn document (n = 4)

Wrong focus (n = 59)

Wrong patient population (n = 1)

Wrong setting (n = 7)

Wrong study design (n = 4)

References removed **(n = 514)**

Duplicate studies (n = 181)

Marked as ineligible by date/scope (n = 333)

Studies excluded **(n = 584)**

Reasons: not including findings from the United Kingdom, not concerning age-related publicly-funded healthcare resource allocation and decision-making, full-text not available.

**Screening**

Academic database searches (n=63)

Manual searches: (n=75)

Studies included in review **(n = 138)**

**Included**
